# Gastrointestinal Bleeding From a Transverse Colon Dieulafoy Lesion

**DOI:** 10.7759/cureus.42703

**Published:** 2023-07-30

**Authors:** Xinyu Xie, Jian Qin, Xiaojua Ma, Shanshan Liu

**Affiliations:** 1 Department of Gastroenterology, The Fourth Division Hospital of Xinjiang Production and Construction Corps, Yining, CHN

**Keywords:** case report, transverse colon, endoscopic treatment, gastrointestinal bleeding, dieulafoy lesion

## Abstract

Dieulafoy lesions are an extremely rare disease that can cause gastrointestinal bleeding and colon bleeding. Lower gastrointestinal bleeding caused by a transverse colonic Dieulafoy lesion is extremely rare. This study describes the case of a 68-year-old woman who took oral rivaroxaban for atrial fibrillation and presented with massive lower gastrointestinal bleeding associated with a rare transverse colonic Dieulafoy lesion. Hemostasis was successfully achieved by thermal coagulation and the application of endoscopic hemoclips. Lastly, we reviewed previous literature on the diagnosis and treatment of colonic Dieulafoy disease.

## Introduction

Dieulafoy lesions are an uncommon and potentially serious gastrointestinal bleeding disease. They account for about 1-2% of gastrointestinal bleeding, 80% of which are located in the upper gastrointestinal tract and less than 2% in the colorectum [1]. Lower gastrointestinal bleeding caused by a transverse colonic Dieulafoy lesion is extremely rare [2]. It is usually a small, relatively inconspicuous, temporary, intermittent, or self-limited cause of bleeding and can lead to a diagnostic dilemma that can be very frustrating for both the clinician and patient. Quiescent or intermittent Dieulafoy lesions are likely ignored endoscopically and occasionally misdiagnosed. The treatment of Dieulafoy lesions depends on the patient's clinical symptoms, location of the disease, and available expertise and technology [3]. This case describes an elderly woman with a transverse colonic Dieulafoy lesion accompanied by severe lower gastrointestinal bleeding.

## Case presentation

A 68-year-old woman came to our emergency department complaining of fresh blood stools. She presented with hematochezia seven days after intermittently relieving dark red stool. The patient had a history of hypertension and atrial fibrillation and had been on oral rivaroxaban for one year. She was not using any other medication that may affect coagulation other than rivaroxaban. The patient's blood pressure was around 95/60 mmHg on admission, and physical examination revealed pallor. Her abdomen was soft, with no lump palpable, and free of tenderness or rebound tenderness. On digital rectal examination, bloody stools were noted. She completed the blood routine, biochemistry, coagulation, and other relevant blood tests. Hemoglobin was 55 g/L, hematocrit was 17.9%, platelet count was 194 × 10^9^/L, prothrombin time (PT) was 13.70 seconds, international normalized ratio (INR) was 1.19, and the rest of the assays were within normal limits. She received an infusion of crystal liquid and two units of erythrocyte transfusions, but her blood pressure did not show an upward trend, so she underwent emergency colonoscopy on the second day of hospitalization. Endoscopy revealed a significant amount of blood clots in the left hemicolon and transverse colon and yellow feces in the ascending colon. However, there were no identifiable sources of bleeding, such as colorectal cancer, diverticulum, arteriovenous malformation, polyp, or colitis.

**Figure 1:**
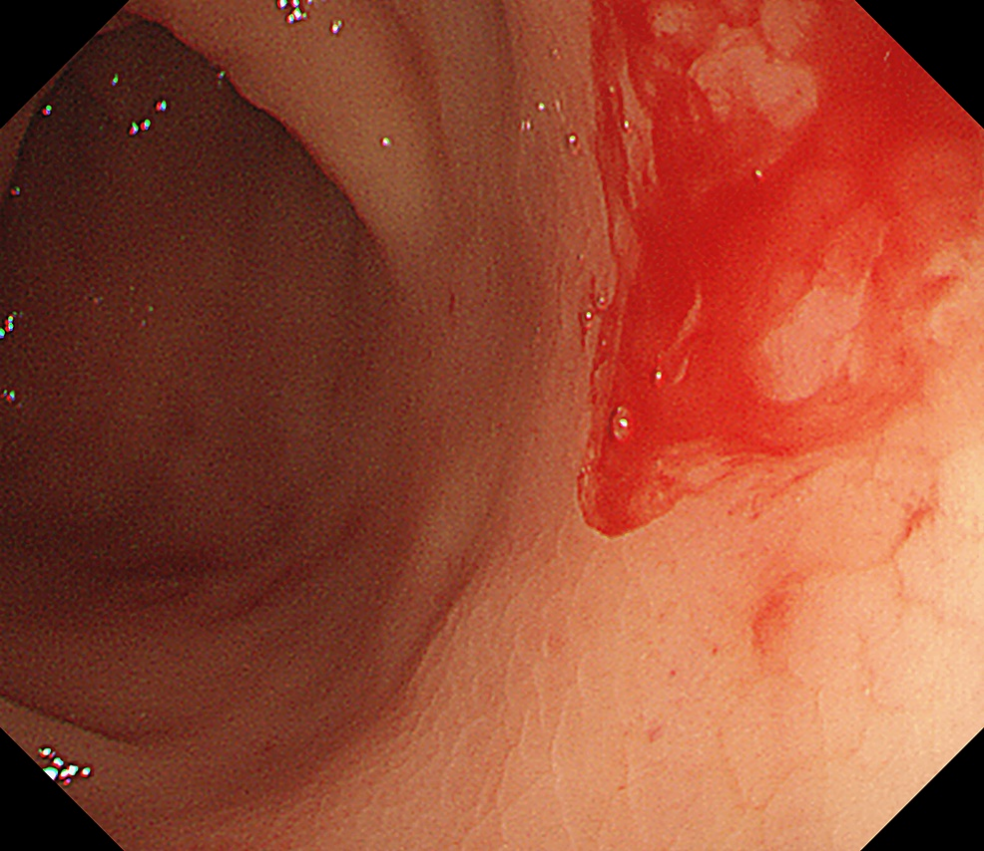
Active transverse colon bleeding

**Figure 2:**
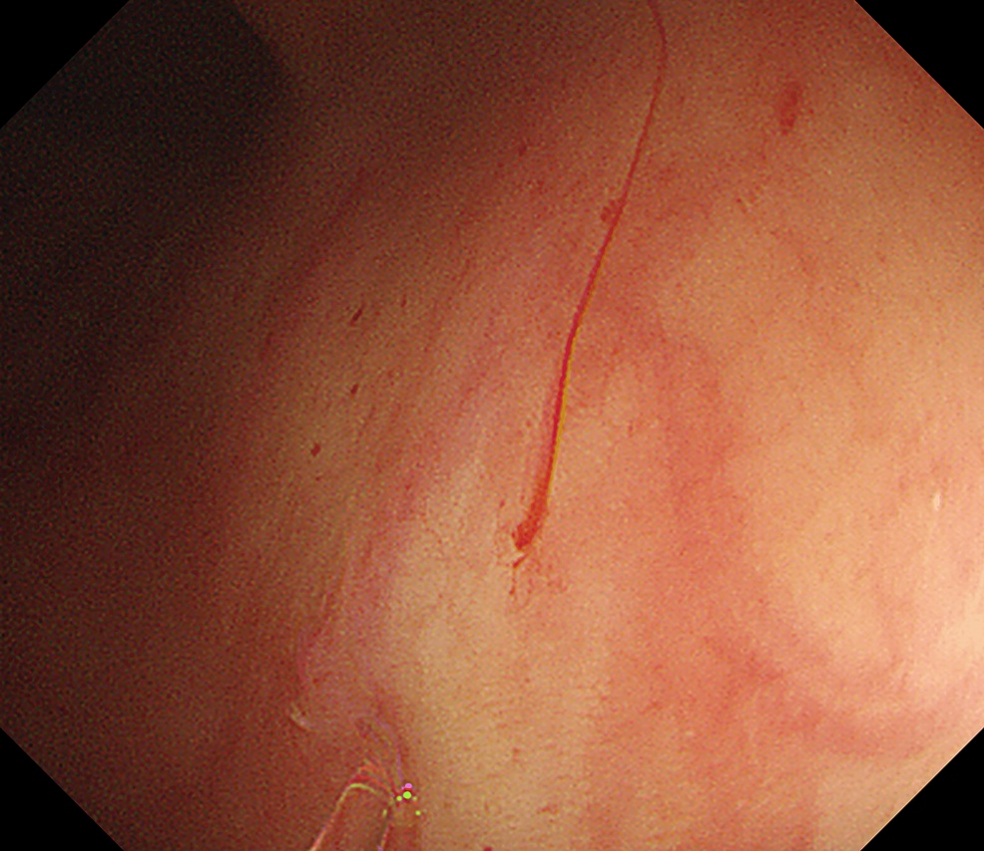
Irrigation observation of Dieulafoy lesion bleeding on white-light imaging

**Figure 3:**
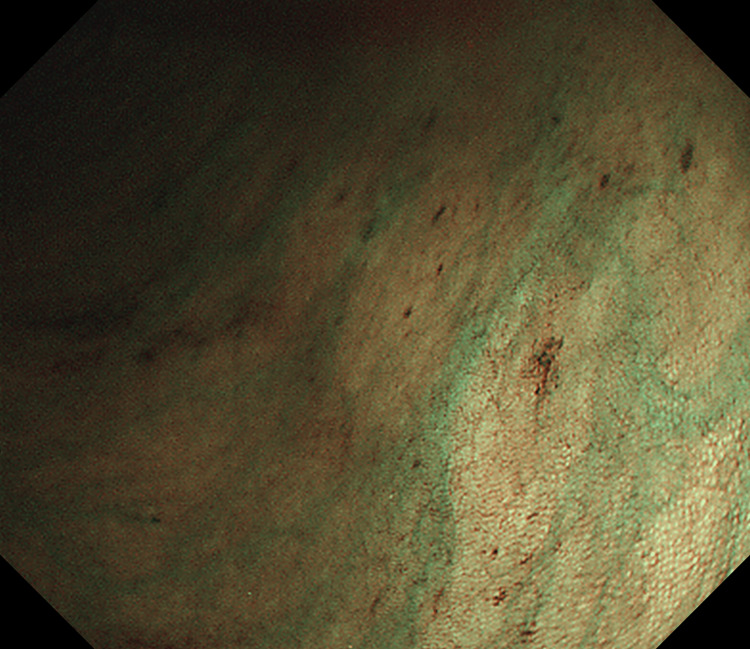
Observation of Dieulafoy lesion on narrow-band imaging

**Figure 4:**
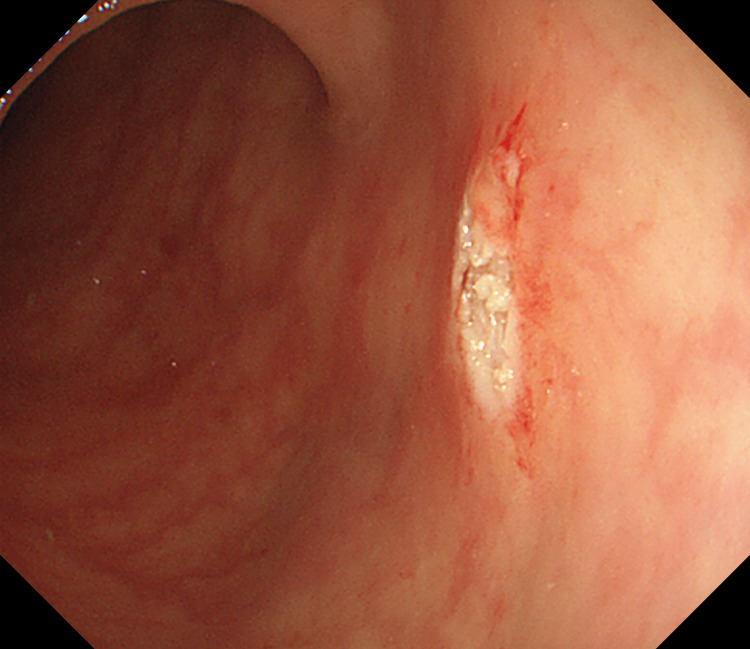
Thermal coagulation was successfully performed

**Figure 5:**
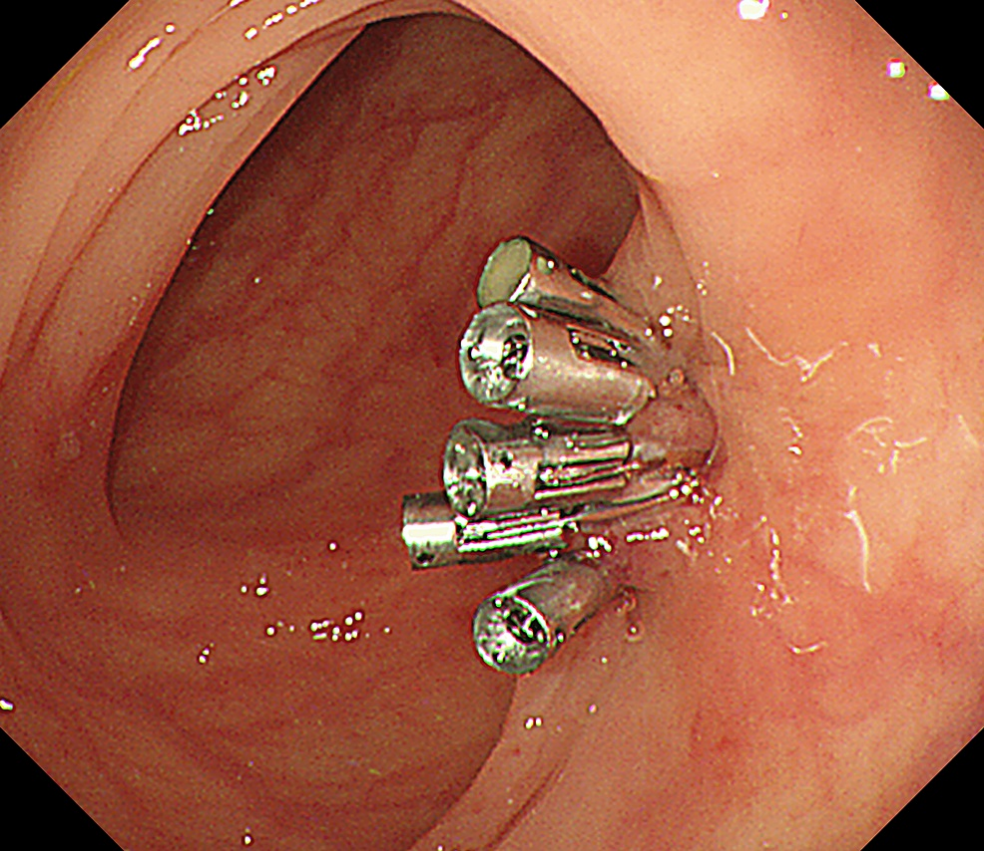
Five endoclips applied for hemostasis

After a careful examination following the removal of the densely adherent blood clots, an actively bleeding site was localized near the proximal transverse colon (Figure 1). After repeated rinsing with room-temperature water, a careful observation revealed active bleeding from a minuscule vessel within a minute mucosal defect, prompting the diagnosis of a Dieulafoy lesion in the colon (Figure 2). When we were preparing for further treatment, the lesion suddenly stopped bleeding. We looked for and compared the shape of blood vessels around the lesion again and determined it with narrow-band imaging: a minute mucosal defect with normal surrounding mucosa and no inflammation (Figure 3). Hemostasis was successfully achieved by thermal coagulation (monopolar, soft coagulation, power 80 w) (Figure 4) and the application of endoscopic hemoclips (Figure 5). After the operation, she received two units of erythrocyte transfusions again, and the hemoglobin level rose to 76 g/L. After five days of inpatient observation, without additional therapeutic intervention, her blood pressure remained at 120/80 mmHg, and there was no bleeding. The patient was discharged. Follow-up for seven days showed no signs of rebleeding.

## Discussion

Dieulafoy lesions have been reported in 1-2% of gastrointestinal bleeding [3]. They were originally described in 1884 by Dr. M.T. Gallard as “miliary aneurysms” of the stomach [4]. Fourteen years later, the lesions were christened after the French surgeon Paul Georges Dieulafoy, who, in 1898, designated them as "exulceration simplex" [5]. Today, Dieulafoy lesions are defined as a persistent submucosal arterioles of abnormal calibre, 1-3 mm in diameter, approximately 10 times the average caliber of mucosal capillaries, which do not decrease when they approach the mucosa. Histologically normal vessels without vasculitis, atherosclerosis, or aneurysms [6] are characterized by subintimal arterial fibrosis and no inflammation at the mucosal defect margins [7].

These lesions are typically present in the upper gastrointestinal tract, and extragastric Dieulafoy lesions are extremely rare [1]. It is diagnosed endoscopically, as our case shows: densely adherent blood clots and active bleeding from a minute mucosal defect; in the quiescent phase: the mucosal defect is extremely small, with normal surrounding mucosa and no inflammation. In 1985, Barbier et al. [8] first described Dieulafoy-like diseases in the colon. It was initially thought that colonic Dieulafoy lesions predominated in the right hemicolon, but further reports have shown that they are present all over the colon [2]. In 1993, the first report of effective endoscopic treatment of colonic Dieulafoy lesions was published by Abdulian et al. [9]. Dieulafoy lesions in the transverse colon are extremely rare, with only a few cases reported [2,10-13]. These lesions are commonly found in older male patients with an average age of onset greater than 50 years [14]. Patients on anticoagulation are at greater risk of bleeding, as in our case. Other etiologies of Dieulafoy lesions include stress, alcoholism, fecalomas, and respiratory or cardiac failure. More than 80% of patients suffering from Dieulafoy lesions had related conditions, according to previously published studies [15].

The mechanism of injury rupture in Dieulafoy lesions remains unclear. One theory suggests that minimal trauma and ischemia to the overlying mucosa result in attenuation of the mucosal wall, leaving it particularly susceptible to erosion and bleeding [6]. Endoscopy, angioembolization, and surgical resection are the main treatments for Dieulafoy lesions. Surgery, associated with mortality rates of up to 80%, remained the primary treatment for Dieulafoy lesions until 1990 [3]. With improvements in endoscopic techniques, the treatment of gastrointestinal bleeding associated with Dieulafoy disease has shifted from surgery to endoscopy. The rate of diagnosis of Dieulafoy lesions was increased to 70% in patients by improving the accuracy of imaging, and the rate of successful treatments rose to 90% [16]. One study found that the initial hemostasis rate was similar between epinephrine injections and hemoclips. However, the recurrence rate was lower with hemoclips [17]. Another study also found similar results, with endoscopic clips' rebleeding rate of 8.3% versus 33% for injection therapy alone [18]. Endoscopic hemostasis of Dieulafoy lesions is recommended by the European Society of Gastrointestinal Endoscopy; such endoscopic techniques include thermal coagulation, mechanical hemostasis with hemoclips or band ligation, and diluted epinephrine injection combined with thermal or mechanical therapy [19]. Hemostasis with hemoclips or band ligation is an effective method for treating hemorrhagic Dieulafoy disease, with similar rates of initial hemostasis and recurrent bleeding [20]. Combined thermal and mechanical techniques are superior to injected monotherapy [14]. In the event that endoscopic intervention fails or is not technically possible, consideration should be given to transcatheter angiographic embolization or surgery [19].

## Conclusions

This case shows the significance of considering rare diseases and careful endoscopy in the differential diagnosis of low gastrointestinal bleeding. The risk of gastrointestinal bleeding in our patient increased due to therapeutic anticoagulation. Combined endoscopic therapy is considered the ideal management for Dieulafoy lesions of the colon, with better hemostatic effect, lower risk of rebleeding, and lesser complications. Our patient achieved hemostasis by a combined endoscopic treatment without rebleeding, at one week post-colonoscopy. This case expands the diagnostic considerations for lower gastrointestinal bleeding by adding Dieulafoy lesions in the transverse colon, a rare source of intermittent bleeding that can be treated endoscopically.
